# Multimodal assessments of Zika virus immune pathophysiological responses in marmosets

**DOI:** 10.1038/s41598-018-35481-6

**Published:** 2018-11-20

**Authors:** Fok-Moon Lum, Wei Zhang, Kheng-Choon Lim, Benoit Malleret, Teck-Hui Teo, Jun-Jia Koh, Kuan J. Lee, Tze-Kwang Chua, Yiu-Wing Kam, Wearn-Xin Yee, Isaac Huen, Jeslin J. L. Tan, Siti Naqiah Amrun, Bhanu Prakash KN, Patrick J. Cozzone, Laurent Renia, Philip T. H. Lee, Lisa F. P. Ng

**Affiliations:** 1 Singapore Immunology Network, Agency for Science, Technology and Research (A*STAR), Singapore, 138648 Singapore; 2 Biological Resource Centre, Agency for Science, Technology and Research (A*STAR), Singapore, 138668 Singapore; 3 Singapore Bioimaging Consortium, Agency for Science, Technology and Research (A*STAR), Singapore, 138667 Singapore; 4 Department of Diagnostic Radiology, Singapore General Hospital, Singapore, 169608 Singapore; 5 Department of Microbiology and Immunology, Yong Loo Lin School of Medicine, National University of Singapore, Singapore, 117545 Singapore; 6 National Institute of Health Research, Health Protection Research Unit In Emerging and Zoonotic Infections, Liverpool, L69 3GL UK; 7 Institute of Infection and Global Health, University of Liverpool, Liverpool, L69 7BE UK; 8 Department of Biochemistry, Yong Loo Lin School of Medicine, National University of Singapore, Singapore, 117596 Singapore

## Abstract

Animal models that recapitulate the human pathophysiology have been developed as useful research tools. Although laboratory mice are widely used, they are phylogenetically “distant” to humans. New world monkeys, such as the common marmoset (*Callithrix jacchus*) have steadily gained prominence. In this report, marmosets are explored as an alternate *in vivo* model to investigate infection and immunity of Zika virus (ZIKV). Multimodal platforms, including ultrasound and magnetic resonance imaging (MRI), flow cytometry, and multiplex microbead immunoassays were established to comprehensively decipher immune responses and pathophysiological outcomes. While ZIKV-infected marmosets had detectable ZIKV RNA load in various body fluids, animals did not develop any observable lesions in their testes and brains as shown by ultrasound and MRI. Immune-phenotyping detected differences in the numbers of B cells, CD8+ T cells and HLADR+ NK cells during the first two weeks of infection. Neutralizing ZIKV-specific antibodies were elicited to high levels and targeted epitopes in the E protein. This study presents a *one-stop-shop* platform to study infection and pathophysiology in marmosets. While marmoset-specific research tools are being refined, the research values of these animals present them as a good model for immune-based therapies.

## Introduction

Over the past decades, the spread of emerging and re-emerging infectious diseases across the globe^1^ highlighted the continuous need for advanced investigative tools to accelerate the understanding of these diseases. While basic pathological features of most diseases can be probed *in vitro* using immortal cell lines and primary human cells, cell culture lacks the complexity to model the dynamics of physiological host-pathogen interaction. Rather, the use of pertinent *in vivo* animal models that recapitulate the human pathophysiology can provide further insights into the disease pathogenesis and concomitantly serve as a platform to test the efficacy of potential therapeutics^2^.

The laboratory mouse is the most commonly used animal in studying infectious diseases and immunology^3^. However, these mice are physiologically distinct from humans and great caution has to be exercised in the extrapolation of the results from mouse to man^4,5^. In contrast, non-human primates (NHPs) are both phylogenetically and anatomically closer to humans^6^. Amongst the NHPs, the rhesus macaque, *Macaca mulatta*, and long-tailed macaque, *Macaca fascicularis*, are Old World monkeys that are most commonly used in biomedical research^7^. A viable alternative would be the New World Monkeys, which, despite being difficult to handle, are smaller in size and pose a much lower zoonotic risk, thereby making them attractive alternatives to their larger counterparts^8^. Species include the common marmoset (*Callithrix jacchus*), the squirrel monkey (*Saimiri* sp.), the owl monkey (*Aotus* sp.), and the titi monkey (*Callicebus cupreus*)^8^. To maximize the amount and quality of information that can be derived from NHP models of infectious diseases, sophisticated and advanced technology must be harnessed.

Zika virus (ZIKV) is an arbovirus that is transmitted primarily via the bites of infected *Aedes* mosquitoes. Although ZIKV infection is rarely fatal^9,10^, severe neurological complications have been reported in the 2015–2016 ZIKV outbreaks. These include Guillain-Barré Syndrome (GBS), encephalitis, meningoencephalitis, acute myelitis in adults^11–13^, as well as severe infant microcephaly^9,14,15^. NHP models for ZIKV have been developed over the years^16,17^. However, majority of them were performed with the macaques^18–38^. Importantly, marmosets have been reported as reservoirs of ZIKV^39^. In this study, to establish investigative tools in disease monitoring in marmosets, ZIKV was used. Comprehensive multiple investigative platforms were setup to assess the immune responses of ZIKV-infected animals: (1) Immune-phenotyping was performed with flow cytometry, utilizing a novel panel of cross-species-specific antibodies; (2) cytokine profiling was carried out with a non-human primate microbead-based immunoassay; and (3) humoral response was characterized with both virion- and peptide-based immunosorbent assays. Pathophysiological changes in the brain and testes were also assessed using non-invasive magnetic resonance imaging (MRI) and ultrasound.

## Results

### Virus infection of marmosets and detection of viral RNA in various body fluids

**Figure 1:**
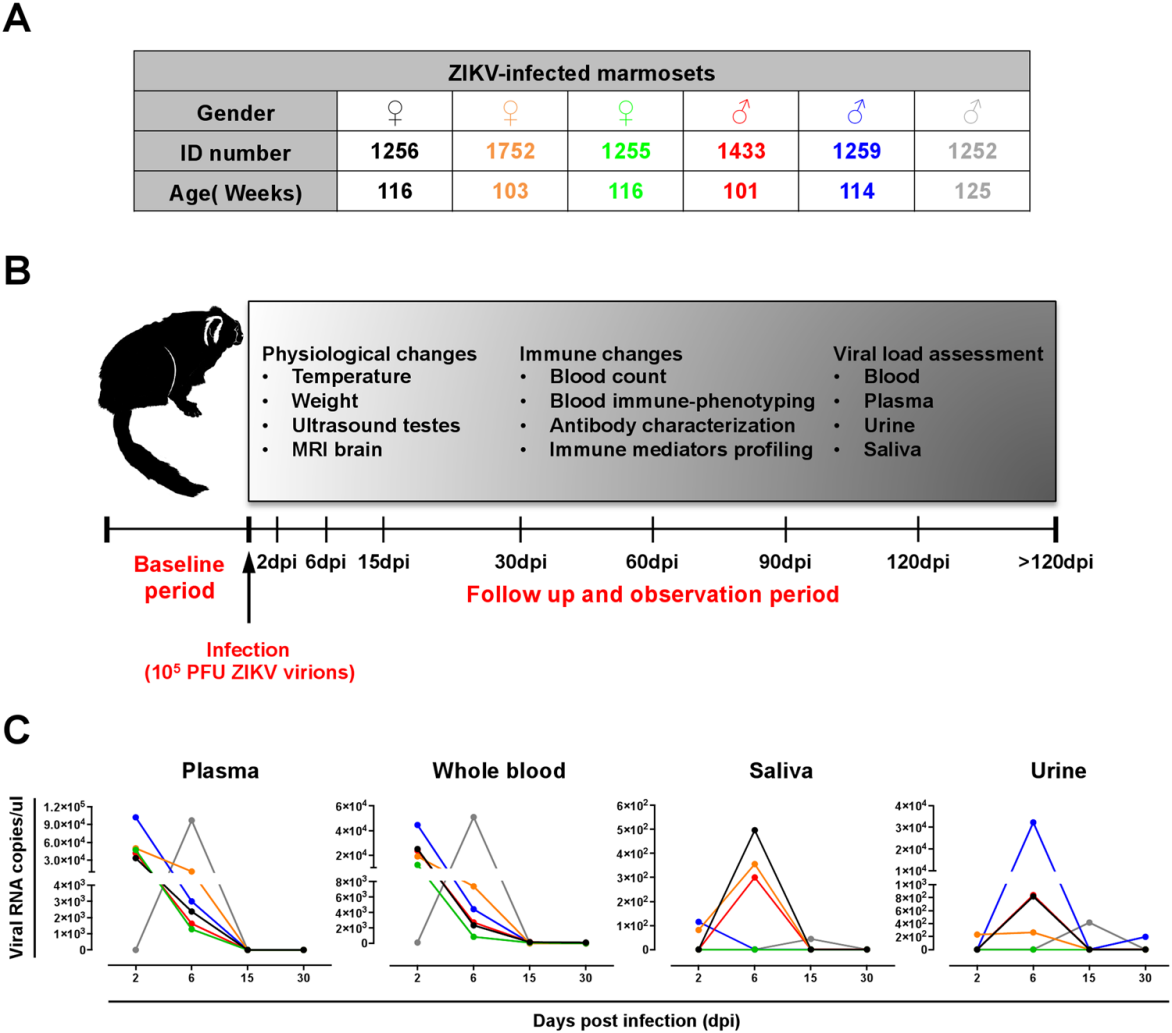
Schematic diagram of infection timeline and identification of infected marmosets. (**A**) A total of six marmosets, aged between 101–125 weeks were used in the study. (**B**) Marmosets were infected with 10^5^ PFU of ZIKV and observed at the stated time-point: 2, 6, 14, 30, 60, 90, 120 and > 120 days post-infection (dpi). Viral load and physiological and immune changes were assessed at each follow up. (**C**) ZIKV RNA load assessment in plasma, whole blood, saliva and urine up to 30 dpi with a RT-PCR targeting the ZIKV NS5.

Six adult marmosets (three males and three females) aged between 101–125 weeks old were selected for this study (Fig. 1A). An initial “baseline” 3-week period was carried out to optimize experimental procedures (Fig. 1B). Virus infection was performed with inoculation of 1E5 PFU of ZIKV virions via the saphenous vein. ZIKV-infected marmosets were observed at stated time-points over 4 months (Fig. 1B). Physiological and immune changes were assessed at selected time-points. Marmosets were also assessed for the presence of ZIKV RNA load in the various body fluids (Fig. 1B).

Corroborating the findings in ZIKV-infected patients^40^, ZIKV RNA load was detected in marmosets’ whole blood, plasma, saliva and urine specimens. Virus detection was highest in the plasma, followed by whole blood samples, with the first traces of ZIKV RNA detected as early as 2 days post-infection (dpi). Detectable levels started to decrease from 6 dpi to trace levels of ~100 copies/ul at 15 dpi and went below detection limit by 30 dpi (Fig. 1C). In the urine and saliva specimens, low viral RNA load was observed at 2 dpi and peaked at 6 dpi (Fig. 1C). Although no gender-bias was observed in viral load, variation across the ZIKV-infected marmosets in terms of absolute quantities, especially in the urine and saliva samples was observed (Fig. 1C).

### Clinical and pathophysiological changes in ZIKV-infected marmosets

ZIKV-infected marmosets showed mild clinical signs during the first week of infection and exhibited no further disease complications for the remaining study period. However, all five ZIKV-infected marmosets were observed to have some soft dark stools from day 1–7 dpi, which was resolved by the second week. Although animals appeared slightly lethargic during the first week of infection, they were alert and responsive. None of the animals showed any signs of joint pain, limping or conjunctivitis. These symptoms were consistent with a mild viral infection^41,42^. These animals also did not experience any changes in temperature and weight (Fig. S1). Similarly, non-infected marmosets (3 females and 2 males) (Fig. S1A), also did not experience any changes in body weight and temperature (Fig. S1B–E).

**Figure 2:**
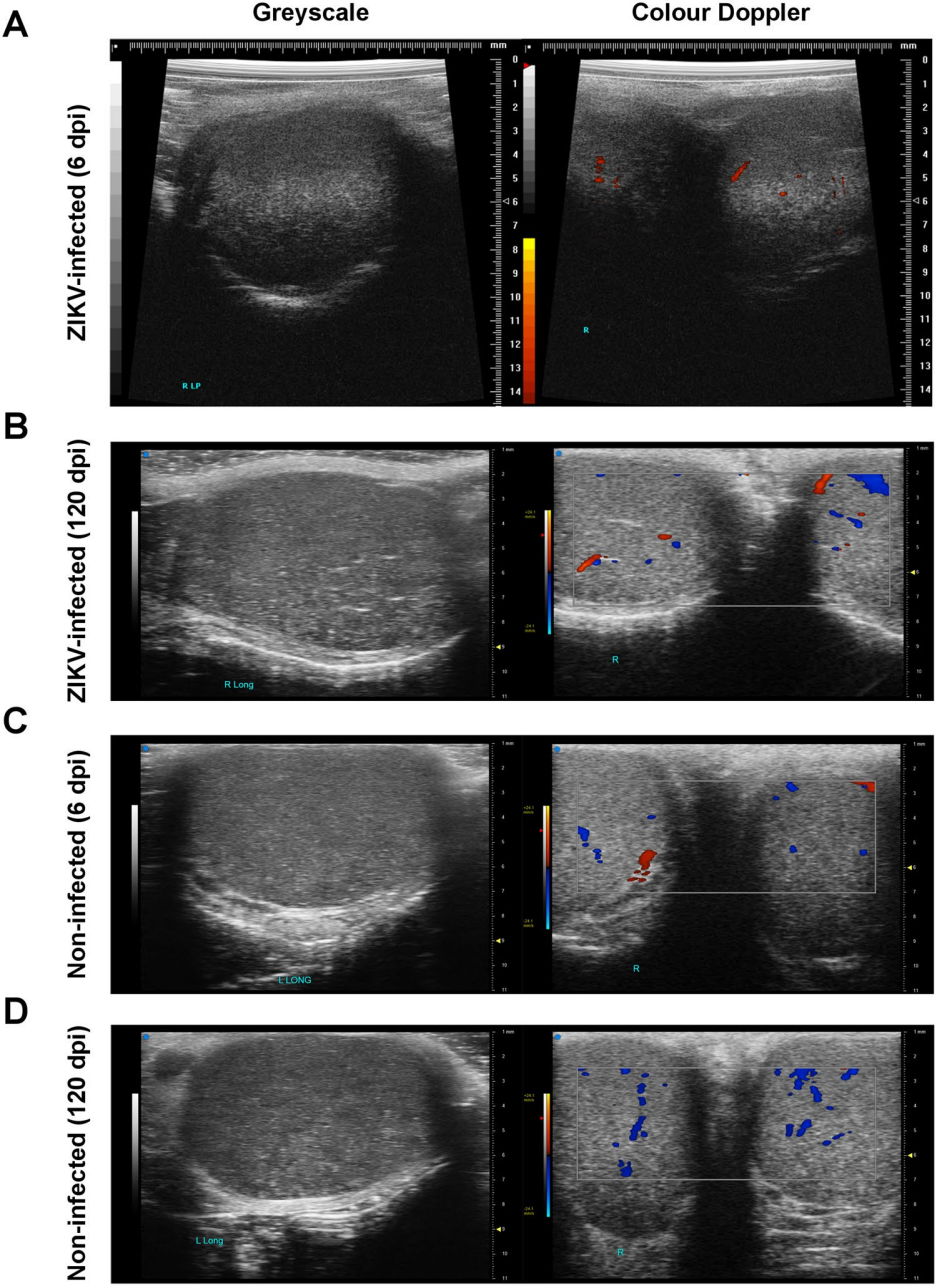
Ultrasound testes images. Illustrations depicted in the left column are shown as greyscale images, while those in the right column are colour Doppler images of both testes at the midline along the transverse plane. The grey scale images show normal testicular echotexture. Colour pixels represent blood flow with different colour representing in-plane/out-plane flow direction. Images shown are representative of (**A**,**B**) ZIKV-infected or (**C**,**D**) non-infected marmosets obtained at (**A**,**C**) 6 or (**B**,**D**) 120 dpi. No abnormal flow is evident on colour Doppler to suggest inflammation.

It was reported that ZIKV could infect testicular and neural cells^43,44^, thus both testes and the brains of the marmosets were evaluated in this study. Fortunately, there were no pathological sonographic findings on grey scale and Doppler evaluation of both ZIKV-infected and non-infected groups (Fig. 2). Images indicated an absence of inflammation in the testes during both acute (6 dpi; Fig. 2A and C) or post-acute (120 dpi; Fig. 2B and D) disease phase. Testicular volume derived manually with a vernier caliper (Fig. S2A–C), and with ultrasound imaging (Fig. S2D–F) showed no statistical significance between the two groups of animals (P = 0.09, 0.18 respectively) and between the two groups with the passage of time (P = 0.11, 0.47 respectively) (Fig. S2C and S2F).

**Figure 3:**
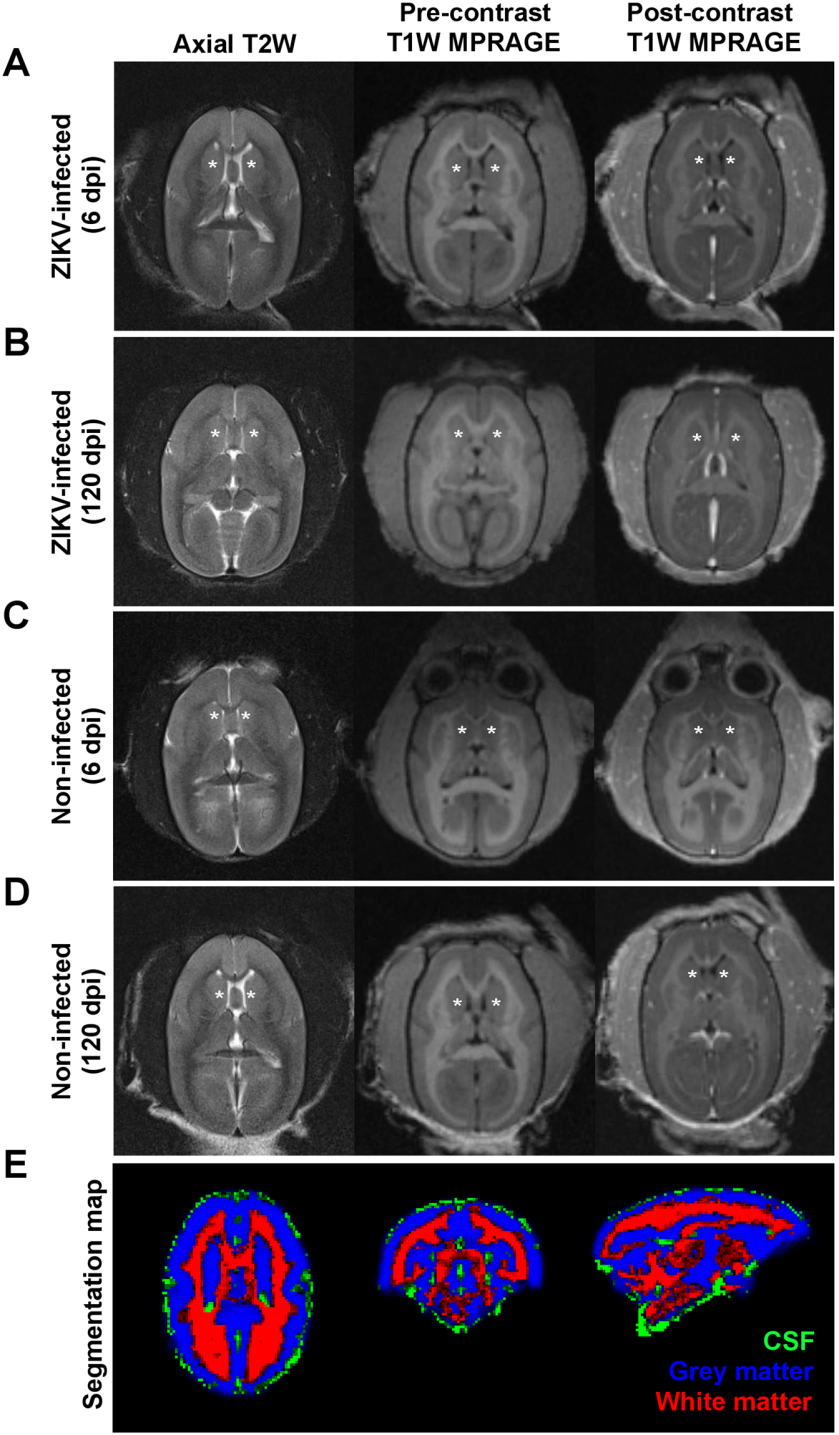
MRI brain images. (**A**–**D**) From left to right columns, axial T2-weighted fast spin-echo, pre- and post-contrast T1-weighted MPRAGE images of the marmoset brain at the level of the basal ganglia with asterisk (*) denoting the caudate nucleus. These represent (**A**,**B**) ZIKV-infected or (**C**,**D**) non-infected marmosets obtained at (**A**,**C**) 6 or (**B**,**D**) 120 dpi. No focal abnormality or pathological enhancement is seen in the ZIKV-infected brain. (**E**) Segmentation map of the marmoset brain for white, grey matter and cerebrospinal fluid (CSF).

Given the small size of the marmosets, optimization for the MRI was needed (see Materials and methods), and MRI on the brains of ZIKV-infected and non-infected animals did not reveal any focal abnormality or significant finding during acute (6 dpi; Fig. 3A and C) or post-acute (120 dpi; Fig. 3B and D) disease phase. The calculated brain volumes (Fig. 3E and Fig. S2G–I) also did not show any significant differences between the two groups of animals (P = 0.28) and between the two groups with the passage of time (P = 0.83) (Fig. 2I).

### Changes in profile of immune cells during post ZIKV infection

**Figure 4:**
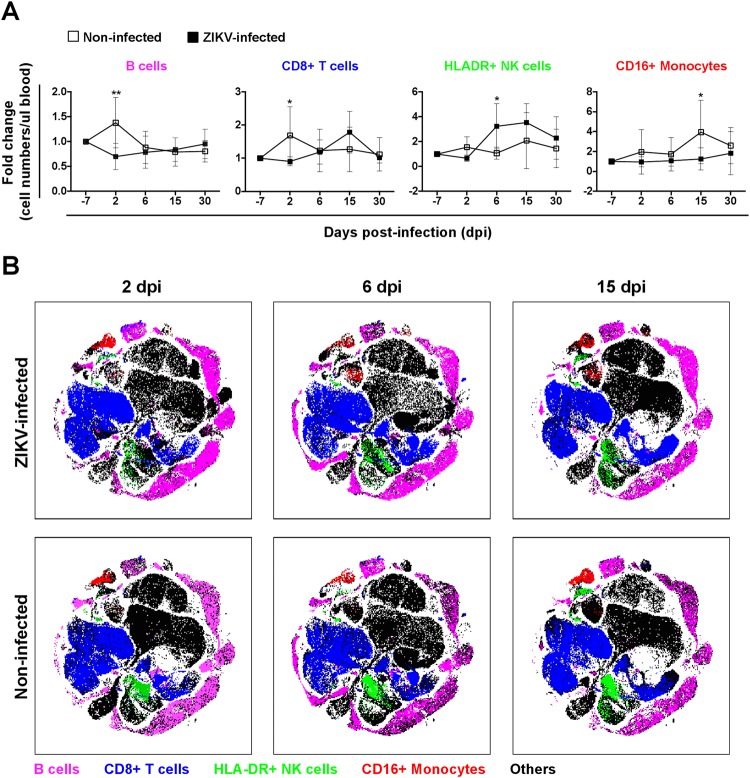
Immune-phenotyping of ZIKV-infected marmoset whole blood. Immune cell changes (up to 30 dpi) were determined at each time-point with flow cytometry. (**A**) Four main immune subsets (B cells, CD8+ T cells, HLA-DR+ NK cells and CD16+ monocytes) which displayed significant differences between ZIKV-infected (n = 6) and non-infected (n = 5) marmosets are shown. Data is presented as fold change relative to baseline (−7dpi) value. (**B**) Flow cytometry data were further visualized by tSNE to identify the specific differences (based on spatial distribution) between the immune subsets shown in (**A**). Data are plotted as mean ± SD. *P < 0.05 by non-parametric Mann Whitney test, two tailed.

As ZIKV RNA load was cleared within 30 days (Fig. 1C), specific peripheral immune cell changes within this period were studied (Fig. S3; see supplementary discussion online). Notably, of the 15 immune subsets identified (Fig. S4), only the B cells, CD8+ T cells, HLADR+ NK cells and CD16+ monocytes exhibited significant differences between the ZIKV-infected and non-infected groups (Fig. 4A). ZIKV infection resulted in lower B-cell and CD8+ T-cell numbers at 2 dpi, higher HLADR+ NK cells at 6 dpi and reduced CD16+ monocytes at 15dpi (Fig. 4A). However, it would be crucial to note that in the non-infected marmosets, some fluctuations were observed in their peripheral immune cell numbers, which may confound findings presented in Fig. 4A. Nonetheless, to further define any phenotypic differences within these immune subsets, data was interpreted with tSNE, a method that conserves the high dimensionality of the data and allows for easy visualization of the data on two dimensions^45^. Despite the differences in cell numbers (Fig. 4A), no further phenotypic differences were observed between the non-infected and ZIKV-infected marmosets (Fig. 4B).

### ZIKV infection elicits neutralizing anti-ZIKV antibodies

**Figure 5:**
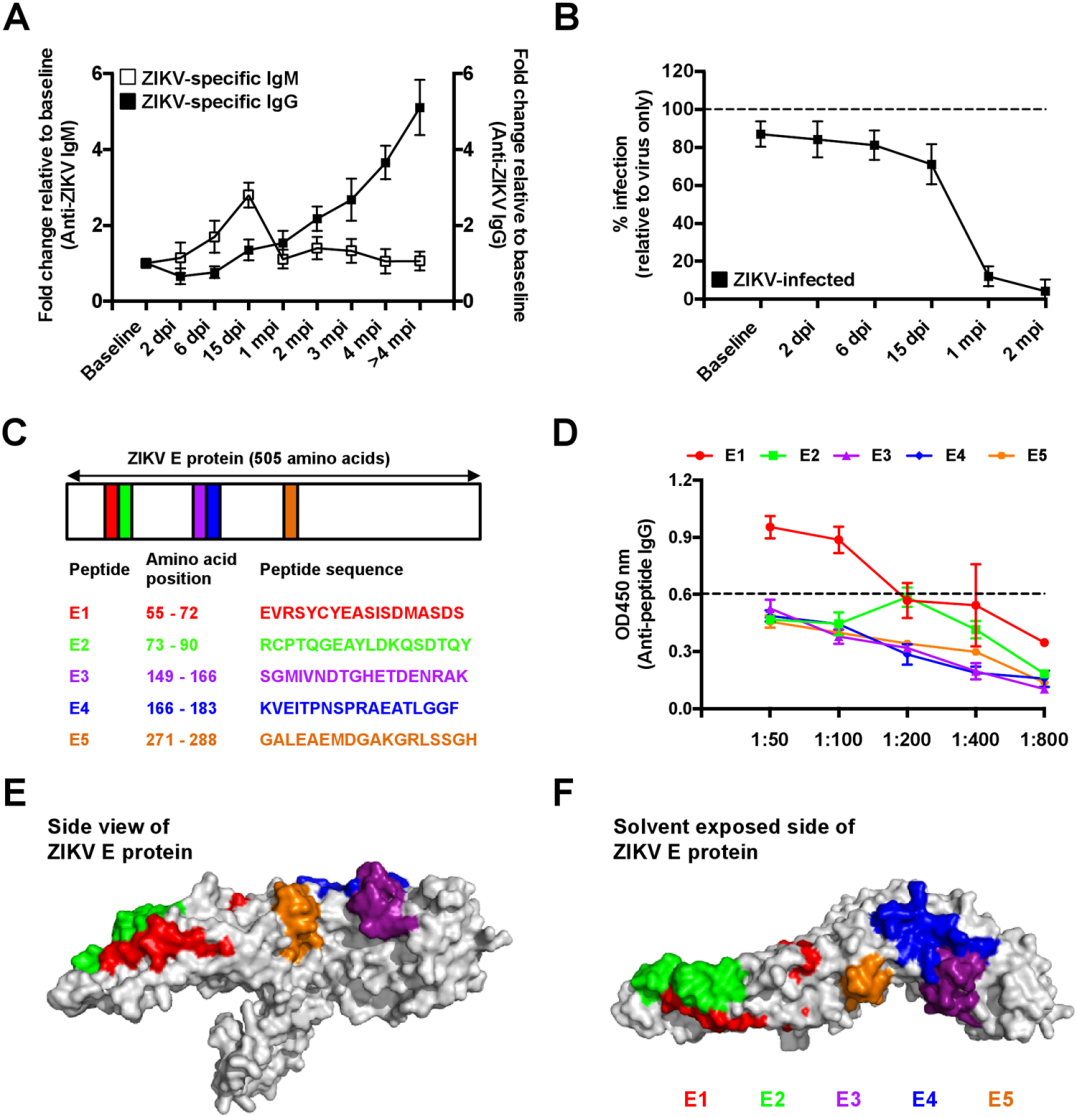
Specificity of ZIKV antibodies. (**A**) Production kinetics of ZIKV-specific IgM and IgG antibodies over the course of study were profiled using ZIKV-specific ELISA. Individual samples were assayed in duplicates. Data is presented as fold change in antibody titers relative to baseline levels. (**B**) Neutralization capacity of ZIKV-specific antibodies was determined by the infection (MOI 10) of HEK293T cells in the presence of diluted (1:100) marmoset plasma (n = 6). This is presented as percentage infection relative to the level of infection obtained with virus only at 72 hours post-infection (dotted line). Individual samples were tested in triplicates. Data shown are presented as mean ± SD. (**C**) Positions of screened peptides along ZIKV E protein are illustrated together with their respective peptide sequences. (**D**) The specificity of ZIKV antibodies obtained at >4 mpi was screened against selected ZIKV E protein peptides via ELISA. Screening was done with five different dilutions of pooled plasma samples from all 6 ZIKV-infected marmosets. Samples were assayed in triplicates. Average background signal from mock-infected (n = 5) plasma (tested at 1:50 dilution) is depicted as black dotted line. The positions of the screened peptides on ZIKV E protein are depicted. ZIKV E protein are shown in either (**E**) the side view or (**F**) the top view (solvent exposed side). Epitope modeling was done on the ZIKV E protein structural data retrieved from the Protein Data Bank (PDB) (ID: 5IZ7).

**Figure 6:**
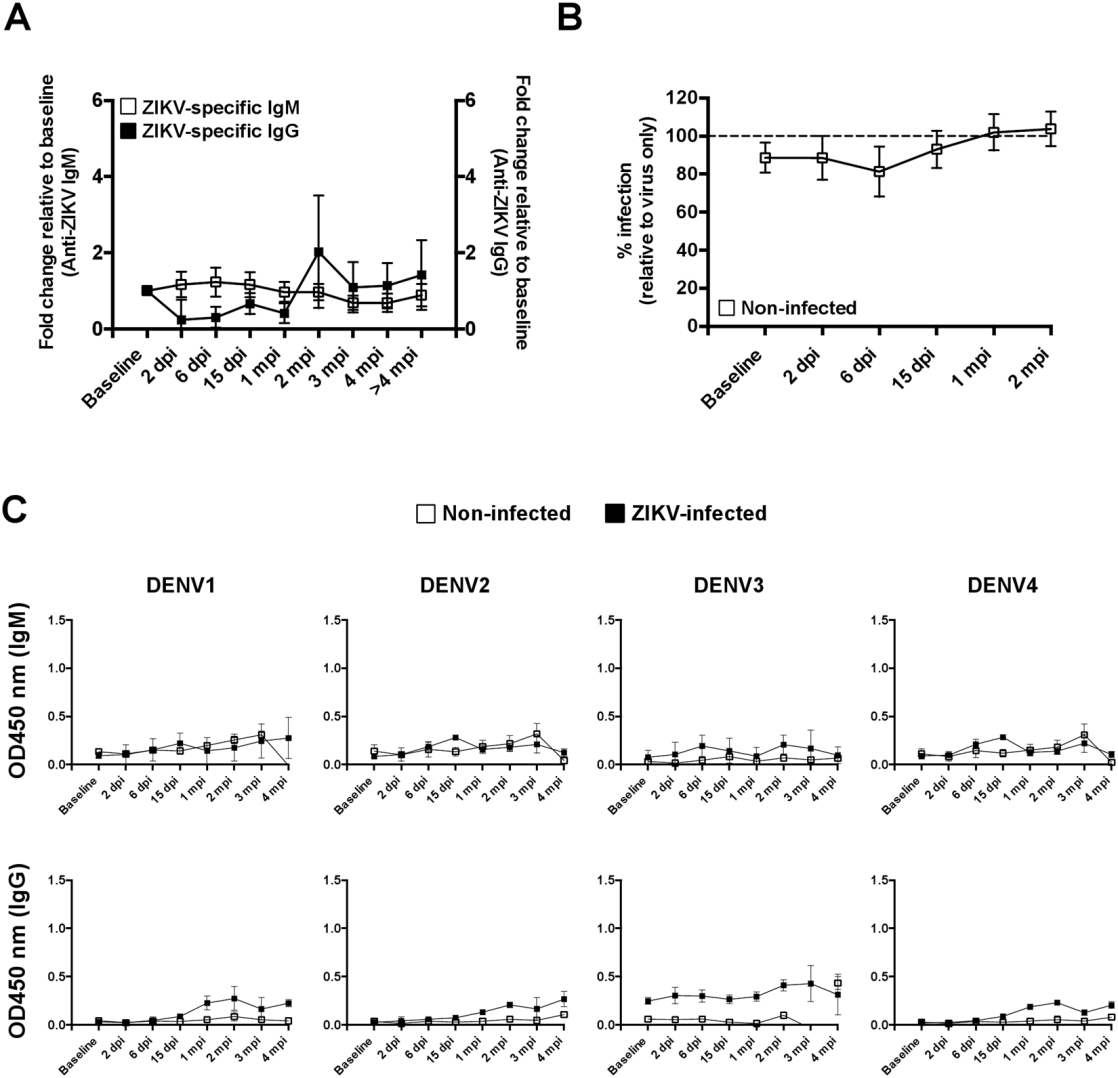
Assessing the cross-reactivity of anti-ZIKV antibodies against DENV. (**A**) ZIKV ELISA datapoints are compiled as line graphs to illustrate the background IgM and IgG signals from non-infected marmosets (n = 5) at the stated time-points. (**B**) The neutralization capacity of non-infected (n = 5) marmoset plasma against ZIKV is depicted. (**C**) DENV (serotypes 1, 2, 3 and 4) ELISA were performed to assess the cross-reactivity of antibodies (both IgM and IgG) from ZIKV-infected (n = 6) and non-infected (n = 5) marmosets. Compiled data from ZIKV-infected and non-infected samples are plotted as mean ± SD and showed minimal cross-reactivity against DENV.

Utilizing a ZIKV virion-based ELISA^46^, anti-ZIKV IgM was detected as early as 2 dpi (Fig. 5A). Consistently, titers of ZIKV-specific IgM peaked at 15 dpi in all six ZIKV-infected marmosets, before declining sharply thereafter (Fig. 5A). The peak of ZIKV-specific IgM response at 15 dpi coincided with the first traces of detectable ZIKV-specific IgG antibodies that continued to increase up to 4 months post-infection (mpi) (Fig. 5A). Plasma obtained from non-infected marmosets was devoid of any ZIKV-specific antibodies (Fig. 6A).

Functionally, efficient *in vitro* neutralization was observed in ZIKV-specific antibodies taken from 15 dpi onwards till 1–2 mpi. (Fig. 5B). The strong inhibition concurs with the production of ZIKV-specific IgG (Fig. 5A), while plasma samples from non-infected animals were non-neutralizing (Fig. 6B). Interestingly, plasma samples from ZIKV-infected marmosets did not cross-react with all 4 serotypes of the DENV virus (Fig. 6C).

### ZIKV-specific antibodies recognizes algorithm-predicted linear epitopes on ZIKV E protein

It was recently reported the ZIKV E protein contains numerous surface-exposed regions that could be targeted by ZIKV antibodies^47^. To determine if ZIKV-specific antibodies induced in the marmosets recognize algorithm-predicted immunogenic linear epitopes within the E protein^47^, five key peptides (labeled as E1, E2, E3 E4 and E5) were selected for the screening (Fig. 5C). Specifically, ZIKV-specific antibodies from > 4mpi were only able to recognize the E1 peptide making it the immune-dominant peptide (Fig. 5D). These five screened peptides were next mapped on to the ZIKV E protein structure shown in either its side view (Fig. 5E) or top view (Fig. 5F) illustrating their positions and level of exposure to the environment.

## Discussion

While larger Old World monkeys continue to remain as an attractive choice to study pathophysiology of diseases^7^, the common marmoset of the New World monkeys could be another interesting consideration^48^. One of the most important features that makes marmosets appealing is their small frame and weight^48,49^ that makes handling easier. In addition, due to the reduced body size, husbandry costs have been estimated to be reduced by approximately 10–15 folds^49^. These animals are also known for their early maturity, great fecundity, short gestation period, which will allows for a quick setup of an in-house breeding colony^49^.

In this report, ZIKV-infected marmosets were free from any visible discomfort or clinical illness despite detection of ZIKV RNA in various body fluids, corroborating earlier studies^50^. These findings also closely reflects ZIKV patients and ZIKV-infected macaques where the majority displayed little or no symptoms^24,35,42,51^, suggesting the marmoset as a good “asymptomatic model”. The presence of detectable viral load in marmoset whole blood, plasma, urine and saliva specimens were also consistent with findings in patients^52^. These results further complement findings from earlier reports where ZIKV RNA load could be detected in serum, saliva and urine specimens for up to 2–3 weeks post-infection^50,53^, albeit with some variations in the levels and persistence of the detected ZIKV RNA. These differences could be due to differences in dosage of the virus inoculum, inoculation route, the strain of ZIKV being used^50,53^, and the physiological states of the animals, as one study was done on pregnant marmosets^53^. Importantly, the detection of ZIKV RNA load in urine and saliva specimens accentuate their potential as non-invasive samples for diagnostic purposes^52^, especially when it is reported that ZIKV RNA load can be detected in urine samples for up to 60 days after symptoms onset in humans^54^.

MRI has been shown to be effective in detecting fetal brain damage in pregnant macaques^18^, while ultrasound is a sensitive technique often used to evaluate for testicular lesion and inflammation. Therefore, non-invasive brain MRI and testes ultrasound procedures were established for longitudinal follow-up. Interestingly, ZIKV infection in marmosets did not reveal any detectable lesions, inflammation or gross structural changes. Inspite of this, it is worthwhile to note that replicating ZIKV has been found in the testes of immunocompromised mice leading to the shrinkage of the organs as well as overall sperm count and motility^55^. ZIKV RNA has also been found in the seminal fluid of ZIKV-infected adult macaques^28^, marmosets^50^ and men^56,57^. ZIKV infection dynamics in the immune-privileged testes of these larger animals should be studied to better understand any long-term impairments of the organ. MRI did not reveal any significant brain abnormalities in ZIKV-infected marmosets as these animals were immunocompetent and like humans, had an intact blood brain barrier. Nevertheless, these techniques are useful as non-invasive follow-up in large animal models.

In terms of immunological responses, ZIKV infection did not lead to significant changes in numbers of the majority of peripheral immune cells. The only notable differences were in the B cells, CD8+ T cells, HLADR+ NK cells and CD16+ monocytes. However, it should also be noted that in non-infected marmosets, changes in peripheral immune cell numbers were similarly recorded, making it hard to interpret if the changes observed were a true consequence of ZIKV infection. Nonetheless, changes in NK cells and B cells have been reported in another marmoset study of ZIKV infection^50^. In ZIKV-infected macaques, induction of activated CD4+ T cells was reported^21,23,29^, which was not observed in the marmosets^50^. In another study, ZIKV-infected rhesus macaques displayed altered levels of peripheral monocytes and dendritic cells^31^. Reasons for this disparate immune responses between marmosets and macaques could be due to the differences in inoculation routes^58^, virus titer or ZIKV isolates used. In ZIKV-infected humans, a transient decrease in numbers of most peripheral immune subsets was detected during the acute phase of the disease (<7 days post-illness onset), before recovering^40^. In the quantification of soluble immune mediators present in the blood, a lack of significant differences were observed after ZIKV infection (Supplementary Table 2). Further optimization would be required as the NHP microbead kit used in this study may not be the most specific for marmosets, thus resulting in the lack of quantifiable differences during the post infection phases. It would be interesting to point out that similar observation was earlier reported in another cohort of ZIKV-infected marmoset^50^, where majority of the immune mediators assayed were not affected by ZIKV infection. This contrasts findings reported in human patients, where levels of soluble immune mediators present in their plasma were significantly modulated by the infection^40,59^. This further accentuates the urgent need to develop a more sensitive and marmoset-specific microbead assay.

Elicitation of robust neutralizing ZIKV-specific antibodies indicates active infection of the marmosets, with high titers of ZIKV-specific IgG being detected up to at least 4 mpi. While it was not performed in this report, the elicited ZIKV-specific IgG should be able to cross-protect against a re-infection by another ZIKV strain^50^. In fact, heterologous ZIKV infection in marmosets further boosted the humoral response^50^. Importantly, these antibodies exhibited minimal cross-reactivity to DENV. Despite inefficient neutralization, ZIKV-specific IgM could be a good diagnostic measure of acute ZIKV infection because these IgM do not cross-react with DENV. The ZIKV E protein is known to be responsible for virus entry^60^ and typically covers the entire viral surface^61^. The specificity of marmoset ZIKV antibodies in targeting the solvent-exposed E1 peptide of the E protein, further emphasizes its importance as a therapeutic or diagnostic target (28). Notably, numerous eptiopes are concealed in the ZIKV E protein, rendering it a major target of ZIKV-specific antibodies from patients as well as murine monoclonal antibodies^61–64^. Nevertheless, screening of other epitopes could also be expanded to include other ZIKV proteins, and such screens should also include ZIKV antibodies derived from other organisms, such as mice and other NHPs. These concerted efforts will allow the identification of universal immunodominant epitopes that could be formulated into peptide-based vaccines^65^.

*In vivo* primate models should not be restricted to macaques only. The idea of using marmosets as the next ideal primate for studying infectious diseases should be further explored as marmoset-specific tools are being developed. This study demonstrated that marmosets could be utilized to understand infection and immunity and present themselves as a feasible platform for evaluating the efficacy of developed immune-based therapies.

## Methods

### Study approval

All animals were housed at the animal facility Biological Resource Centre of the Agency for Science, Technology and Research, Singapore (A*STAR), which is accredited by the Association for Assessment and Accreditation of Laboratory Animal Care, International (AAALAC). Common marmosets aged between 101 to 132 weeks were used in this study. All experimental procedures were approved by the Institutional Animal Care and Use Committee (IACUC No. 161137) of A*STAR, in accordance with the guidelines of the Agri-Food and Veterinary Authority and the National Advisory Committee for Laboratory Animal Research of Singapore. The study was conducted under Animal Biosafety Level 2 (ABSL-2) conditions. All marmosets were screened negative for ZIKV and dengue virus (DENV) by both PCR and serology assays^46,66^ prior to the commencement of the study.

### Housing and husbandry

Animals were pair-housed whenever possible and singly-housed marmosets were given visual, auditory and olfactory access to other marmosets. Marmosets were housed in stainless steel cages of 775 mm × 775 mm × 1750 mm and were always provided with enrichment materials. They received a balanced diet containing commercial pellets (Altromin) and a variety of fresh fruits, eggs and dairy products. The holding room was illuminated with fluorescent lights on a 12 hour light-dark cycle with temperature and humidity maintained at 24–28 °C and 40–70% respectively.

### Virus stock

ZIKV strain (accession KJ776791) used in this study was originally isolated from the French Polynesia outbreak in 2013^67^. The virus was propagated and quantified in Vero-E6 cells (ATCC; CRL-1587) as previously described^68^.

### Virus inoculation

Animals were first sedated with ketamine (10–15 mg/kg) and Atropine (0.025–0.05 mg/kg) via intramuscular injection and maintained on inhalation of anaesthesia using isoflurane provided via a facemask, if sedation was inadequate. 1E5 plaque-forming units (PFU) of ZIKV was prepared in a final volume of 300 μL with PBS and inoculated into the sedated animals via an intravenous catheter placed in the saphenous vein. Immediately following the virus inoculation, 500 μL of saline was infused into the catheter to ensure entry of the entire virus preparation into the circulatory system. Non-infected animals received an equal volume of PBS and saline without virus.

### Physiological assessments

#### Clinical observation

Marmosets were observed for clinical signs and behaviour changes such as reduced appetite, lethargy, weakness, signs of joint pain, limping, conjunctivitis and changes in stools. This clinical assessment was conducted by facility veterinarians daily for the first 14 days of infection, twice weekly up to day 30 then weekly for the remaining period of study.

#### Physiological Measurements

Body weight and temperature of the marmosets were measured on 0, 2, 3, 6, 9, 12, 15, 30, 60, 90 and 120 days post-infection (dpi). Temperature was obtained under light sedation using a rectal thermometer and was taken 10 minutes after sedation with ketamine (10–15 mg/kg) and Atropine (0.025–0.05 mg/kg) via intramuscular injection to ensure that temperature elevation due to stress had equilibrated. Testicular dimensions were measured manually with vernier calipers on 2, 6, 15, 30, 60, 90 and 120 dpi. With this method, testicular volume was determined using the formula for a prolate spheroid: Length × Width^2^ × 0.52^69^.

#### Sample Collection (blood, urine, saliva)

Samples were collected on 2, 6, 15, 30, 60, 90, and 120 dpi. Urine samples were collected from restrained marmosets by gentle manual massage of the lower abdomen into Eppendorf tubes and kept on ice prior to analysis. Blood and saliva collection were performed under light sedation with ketamine (10–15 mg/kg) and Atropine (0.025–0.05 mg/kg) via intramuscular injection. Blood samples were collected into EDTA tubes via venipuncture from femoral veins. Volume collected did not exceed 10% total blood volume weekly. Blood was subsequently spun down at 1200 rpm for 10 minutes for plasma isolation. Saliva samples were collected by swabbing the oral cavity with a sterile swab and placing into 300 μL of RNA*later*® solution (ThermoFisher Scientific).

### Magnetic Resonance Imaging of the Brain

MRI brain was performed on the Siemens Skyra 3T scanner. A total of 11 marmosets (n = 5 non-infected, n = 6 ZIKV-infected) were imaged. Imaging schedule coincided with sample collection schedule. Marmosets were scanned under anaesthesia. The imaging protocol consisted of axial T2-weighted fast spin-echo (FSE), coronal T2-weighted FSE, fluid-attenuated inversion recovery (FLAIR) with fat saturation, readout-segmented diffusion-weighted imaging (DWI)^70^, and pre- and post-contrast T1-weighted MPRAGE. Between 1 to 4 months after the last time point, additional pre-contrast MPRAGE and MP2RAGE images^71^ were also obtained to aid automated volume quantitation (see Supplementary Table 1 for details of scan parameters). The body volume coil was used for transmission and a 4 cm single-channel surface coil for reception, which was positioned over the vertex of the marmoset head and secured by a custom rig. Apparent Diffusion Coefficient (ADC) maps were generated from DWI images. T1 maps were generated from the MP2RAGE data, to which each MPRAGE image was registered for segmentation of gray matter (GM), white matter (WM), cerebrospinal fluid (CSF), and whole brain volume at every time point. Gadolinium contrast was administered intravenously (Magnevist 0.2 mmol/kg). Images were evaluated by a radiologist (co-author K.C.L) for any pathophysiological changes.

### MRI brain volume analysis

Automated image analysis was performed on MRI to yield brain volumes. As conventional brain extraction methods (FSL Brain Extraction Tool^72^) were ineffective due to inhomogeneity arising from sensitivity variations in the surface coil, an atlas-based brain extraction method robust to such inhomogeneity was optimized. This was performed in 3 phases: pre-processing, extraction of brain and segmentation of the grey matter (GM), white matter and cerebrospinal fluid (CSF). During the pre-processing, bias field inhomogeneity correction using the Computational Morphometry Toolkit (CMTK)^73^ (http://nitrc.org/projects/cmtk) was performed. In the second phase, a marmoset brain atlas^74^ was used to extract the brain using linear registration (atlasBREX, FSL 5.0) to yield total brain volume. Subsequently, segmentation into GM, WM and CSF was obtained by deriving a probability map from the calculated T1 map. The masks of GM, WM and CSF obtained from the T1 map were transformed to MPRAGE at each timepoint using linear and nonlinear registration (FSL 5.0) to obtain the final GM, WM and CSF regions.

### Ultrasound Imaging of the testes

Ultrasound was performed on 5 male marmosets (3 ZIKV-infected and 2 non-infected) at the time of MRI using either of 2 machines (Vevo 770, RMV704 probe; Vevo 3100, MX550D probe) from FUJIFILM VISUAL SONICS. Testes were evaluated on greyscale and colour Doppler by a radiologist (co-author K.C.L), with testicular dimensions recorded and volume calculated with the empiric formula of Lambert: Length × Width × Height × 0.71^69^.

### Viral RNA load quantification

RNA samples were extracted from whole blood (40 μL), plasma (40 μL), urine (140 μL) and saliva mixture (140 μL) using the QIAamp Viral RNA Mini Kit (Qiagen) according to manufacturer’s protocols. ZIKV quantification was performed by One-step TaqMan real-time RT-PCR (QuantiTect Probe RT-PCR Kit, Qiagen) with primers and probes targeting ZIKV NS5^75^. Predictive values of this system in human blood and urine have been previously recorded^75^.

### Blood count

Complete blood count (CBC) was performed using 50 µL of whole blood on the Ac·T diff^TM^ Haematology Analyser (Beckman Coulter) according to manufacturer’s instructions. Beckman Coulter 4 C^©^ Plus Tri-Pack Cell Controls (Beckman Coulter) were run before patient specimens to confirm instrument accuracy and precision performance.

### Whole blood staining and flow cytometry

Eighty μL of marmosets’ whole blood (n = 5 non-infected, n = 6 ZIKV-infected) was used for staining. The different immune cell subsets present in whole blood were identified via surface staining with the following antibodies: mouse anti-marmoset CD45 (clone: 6C9, Biolegend), mouse anti-human CD16 (clone: 3G8, Biolegend), mouse anti-human CD14 (clone: M5E2, BD Pharmingen), mouse anti-human CD4 (clone: L200, BD Horizon), mouse anti-human CD3 (clone: SP34-2, BD Pharmingen), mouse anti-human HLADR (clone: L243, BD Biosciences), mouse anti-human CD11c (clone: S-HCL-3, BD Biosciences), mouse anti-human CD20 (clone: H299, Beckman Coulter) and mouse anti-human CD335 (clone: BAB281, Beckman Coulter). Subsequently, cells were fixed and lysis of red blood cells was performed with 1X FACS lysing solution (BD Biosciences). No live/dead staining was performed as the protocol was performed with freshly-drawn whole blood which contains typically very few dead cells. Data was acquired with the BD LSR FORTESSA Flow Cytometer (BD Biosciences). Results were analyzed with FlowJo software version 10.0.7 and visualized with FlowJo plugin, t-distributed stochastic neighbor embedding (tSNE)^45^. Amount of specific immune subset present in per microliter of whole blood is back-calculated by multiplying its percentage of total CD45+ cells by the absolute leucocytes count determined via CBC. Subsequently, fold change differences in calculated cell numbers were expressed relative to the baseline levels.

### Virion- and peptide-based ELISA

Presence and titers of ZIKV-specific antibodies in the plasma of infected marmosets (n = 5 non-infected, n = 6 ZIKV-infected) were determined with a ZIKV virion-based ELISA as previously described^76^. Briefly, purified ZIKV were coated on 96-well microtiter plates (Maxisorp, NUNC). Wells with coated virions were then blocked with 5% skim milk (Nacalai Tesque) in 0.05% Tween-20 (Sigma-Aldrich) PBS for 1.5 hours at 37 °C. Diluted (1:50) heat inactivated marmoset plasma samples were then added and incubated for 1 hour at 37 °C. Secondary antibody, horse-radish peroxidase (HRP)-conjugated goat anti-human IgM (diluted 1:2000) (Invitrogen) or anti-human marmoset IgG (diluted 1:5000) (Invitrogen) was subsequently incubated for 30 minutes at 37 °C before luminescence reactions were developed with 3,3′, 5,5′-Tetramethylbenzidine (TMB) substrate (Sigma-Aldrich). Absorbance was then measured at 450 nm with the Tecan Infinite M200 plate reader (Tecan). Samples were assayed in duplicates. Individual samples were assayed in duplicates. Data obtained (after subtracting signals from non-specific binding and secondary antibody) is presented as fold change in antibody titers relative to baseline levels. DENV virion based Elisa is performed in the same approach. For the peptide-based ELISA, linear epitopes of marmoset ZIKV-specific antibodies were screened as previously described^76^, with synthesized biontinylated peptides (EMC) consisting of 18-mer overlapping peptides generated from the ZIKV Polynesian isolate (KJ776791). Peptides selected for the screening are found on the ZIKV E protein and these region have previously been reported to be targets of ZIKV-specific antibodies of ZIKV-infected patients^47^. Briefly, streptavidin-coated plates (Pierce) were blocked overnight with 0.1% Tween-20, 1% Bovine Serum Albumin (BSA) and 1% Casein (all from Sigma-Aldrich) in PBS. Diluted peptides (1:1000 in 0.1% Tween-20 in PBS (PBST)) were then incubated for 1 hour at room temperature, before heat inactivated pooled marmoset plasma samples (n = 5 non-infected, n = 6 ZIKV-infected) were added at dilutions of 1:50, 1:100, 1:200 and 1:400 and incubated for 1 hour at room temperature. Secondary antibody, horse-radish peroxidase (HRP)-conjugated goat anti-marmoset IgG (diluted 1:2500) (Invitrogen) was then added and incubated for another 1 h at room temperature before TMB substrate (Sigma-Aldrich) was added to develop the luminescence reactions. Thereafter, absorbance was measured at 450 nm with the Tecan Infinite M200 plate reader (Tecan). Pooled plasma sample were assayed in triplicates. Epitope modeling was done on the ZIKV E protein structural data obtained from the Protein Data Bank (PDB) (ID: 5IZ7) and visualized using the PyMOL Molecular Graphics System (http://www.pymol.org). Epitopes mapped on the ZIKV E protein are illustrated in either the side or or top (solvent-exposed) view.

### ZIKV neutralization assay

Neutralizing activity of marmoset ZIKV-specific antibodies (n = 5 non-infected, n = 6 ZIKV-infected) was assessed with infection in HEK293T cells. ZIKV (MOI 10) were mixed (1:1) diluted marmoset plasma (1:50 with serum free DMEM (Hyclone)) and incubated for 2 hours at 37 °C with gentle agitation (350 rpm). Virus-plasma mixtures were then added to HEK293T cells seeded in 96-well plates and incubated for 2 hours at 37 °C. Medium was removed, and cells were replenished with DMEM (Hyclone) supplemented with 10% FBS (Hyclone) and incubated for a further 72 hours at 37 °C. During harvesting, ZIKV-infected HEK293T cells were dislodged by trypsinization and transferred to a V-bottom plate. Cells were first stained with the LIVE/DEAD Fixable Aqua Dead Cell stain Kit (Invitrogen) for 20 minutes before fixation with 4% paraformaldehyde (Electron Microscopy Sciences) for 30 minutes. Cells were subsequently permeabilized with PBS containing 0.2% Tween-20 and incubated for 10 minutes at room temperature. Cells were then stained with the primary rabbit anti-ZIKV NS3 antibody^66^ for 1 hour at 37 °C. This was followed by incubation with the secondary goat anti-rabbit secondary antibody conjugated to fluorescein isothiocyanate (FITC) (Life Technologies) for 1 hour at 37 °C. Stained cells were resuspended in a final volume of 60 μL of PBS and acquisition was performed with the MACSquant® Flow Cytometer (Miltenyi Biotec). Neutralization capacity is calculated as the percentage of infection in HEK293T cells relative to virus-only (positive) control.

### Multiplex microbead immunoassay for cytokine quantification

Cytokine and chemokine levels in in the plasma of marmosets (n = 5 non-infected, n = 6 ZIKV-infected) were measured simultaneously using a multiplex microbead-based immunoassay, ProcartaPlex NHP Cytokine/Chemokine/Growth Factor 37-plex (Thermo Scientific). Additionally, 4 replicates of the plasma of control marmosets (n = 5) were ran on all plates to allow plate normalization. Preparation of reagents as well as immunoassay procedures were performed according to manufacturers’ instructions. Luminex concentrations obtained via the Bio-plex Manager software (using 5 parameter logistic curve fitting) were normalized using median centering to remove potential plate effects. The final adjusted concentrations were then logarithmically transformed to assume normality prior to further data analysis and visualization.

### Statistics

Data analysis for multiplex microbead immunoassay was conducted using one-way ANOVA with post hoc Dunnet’s test to detect for differences between the two groups of animals across the various collections. Results were corrected for multiple testing using the method of Benjamini and Hochberg. Statistical analysis on MRI brain volume, testicular volume derived from vernier caliper and ultrasound was performed using a general linear mixed model with repeated measurements. Marmosets were modeled as random effects, while group (infected, control), follow-up imaging (2, 6, 15, 30, 60, 90,120 dpi) and group by day interaction were modeled as fixed effects after controlling for differences in body weight. The objectives were to determine, (1) if there was an overall statistically significant difference between groups, and (2) if the difference between groups changed significantly with the passage of time (Group x Day interaction). Statistical analysis between ZIKV-infected and non-infected marmosets for changes in peripheral blood cell numbers was determined using non-parametric, Mann Whitney test, two tailed. *P* value < 0.05 is considered significant.

## Electronic supplementary material

Supplementary Information
