# Supplementary material for: Multimodal assessments of Zika virus immune pathophysiological responses in marmosets
Source: Sci Rep. 2018 Nov 20;8:17125. doi: 10.1038/s41598-018-35481-6 (PMC6244230; doi:10.1038/s41598-018-35481-6)
Supplement: Supplementary file 1 — Supplementary Information [file 41598_2018_35481_MOESM1_ESM.pdf]

Scientific Reports

Supplemental Materials

Multimodal assessments of Zika virus immune pathophysiological responses in marmosets

Fok-Moon Lum[1], Wei Zhang[2], Kheng-Choon Lim[3,4], Benoit Malleret[1,5], Teck-Hui Teo[1], Jun-Jia Koh[2], Kuan J. Lee[3], Tze-Kwang Chua[1], Yiu-Wing Kam[1], Wearn-Xin Yee[1], Isaac Huen[3], Jeslin J.L. Tan[1], Siti Naqiah Amrun[1], Bhanu Prakash KN[3], Patrick J. Cozzone[3], Laurent Renia[1,5], Philip T.H. Lee[3]* and Lisa F.P. Ng[1, 6, 7, 8]*

[1]Singapore Immunology Network, Agency for Science, Technology and Research (A*STAR), Singapore 138648, Singapore.

[2]Biological Resource Centre, Agency for Science, Technology and Research (A*STAR), Singapore 138668, Singapore.

[3]Singapore Bioimaging Consortium, Agency for Science, Technology and Research (A*STAR), Singapore 138667, Singapore.

[4]Department of Diagnostic Radiology, Singapore General Hospital, Singapore 169608, Singapore.

[5]Department of Microbiology and Immunology, Yong Loo Lin School of Medicine, National University of Singapore, Singapore 117597, Singapore.

[6]National Institute of Health Research, Health Protection Research Unit In Emerging and Zoonotic Infections, Liverpool, UK.

[7]Institute of Infection and Global Health, University of Liverpool, Liverpool L69 7BE, UK.

[8]Department of Biochemistry, Yong Loo Lin School of Medicine, National University of Singapore, Singapore 117597, Singapore.

Supplementary Discussion

Efforts were first needed to optimize the antibody panel (Fig. S3) for immune-phenotyping. Amongst the selected antibodies, only the anti-CD45 antibody (clone: 6C9) was specific to marmoset. The rest of them were all against the human antigens: CD3 (clone: SP34-2), CD4 (clone: L200), CD20 (clone: H299), CD11c (clone: S-HCL-3), CD14 (clone: M5E2), CD16 (clone: 3G8), CD335 (clone: BAB381) and HLA-DR (clone: L243), corroborating with previous studies [1-4]. During the process of optimization, it was verified that the marmoset CD3 and HLADR antigens could also be detected with clones SK7 and G46-6 respectively, two antibodies that targets the human equivalent. At the same time, numerous clones failed to work – clones HIT8a, RPA-T8 and B9.11 for CD8, clones OKT3 and UCHT1 for CD3, clone RPA-T4 for CD4 and clone MP09 for CD14. The only working anti-CD8 antibody targets specifically the marmoset CD8 antigen (clone: 6F10). However, it was not included in the staining panel, due to a clash of fluorophore wavelength with the anti-CD45 antibody (clone: 6C9). Therefore, in the absence of a working anti-CD8 antibody, the presence of CD8+ T cells was determined with the anti-CD4 and anti-CD3 antibodies, in which the CD8+ T cells would be identified as CD3+CD4-. This was further supported by the comparable percentages of CD8+ T cells obtained with or without the anti-CD8 antibody during staining (Fig. S3B).

References

1. Dunham, J. *et al*. Analysis of the cross-talk of Epstein-Barr virus-infected B cells with T cells in the marmoset. Clin Transl Immunology **6(2)**, e127 (2017).
2. Nelson, M., Loveday, M. Exploring the innate immunological response of an alternative nonhuman primate model of infectious disease; the common marmoset. J Immunol Res **2014**, 913632 (2014).
3. Sato, K. *et al*. Generation of a Nonhuman Primate Model of Severe Combined Immunodeficiency Using Highly Efficient Genome Editing. Cell Stem Cell **19(1)**, 127-138 (2016).
4. Yoshida, T. *et al*. Efficient in vivo depletion of CD8(+) T lymphocytes in common marmosets by novel CD8 monoclonal antibody administration. Immunol Lett **154(1-2)**, 12-17 (2013).

A

Non-infected marmosets					
Gender	♀	♀	♀	♂	♂
ID number	1760	1258	1257	1756	1260
Age(Weeks)	115	111	111	124	132

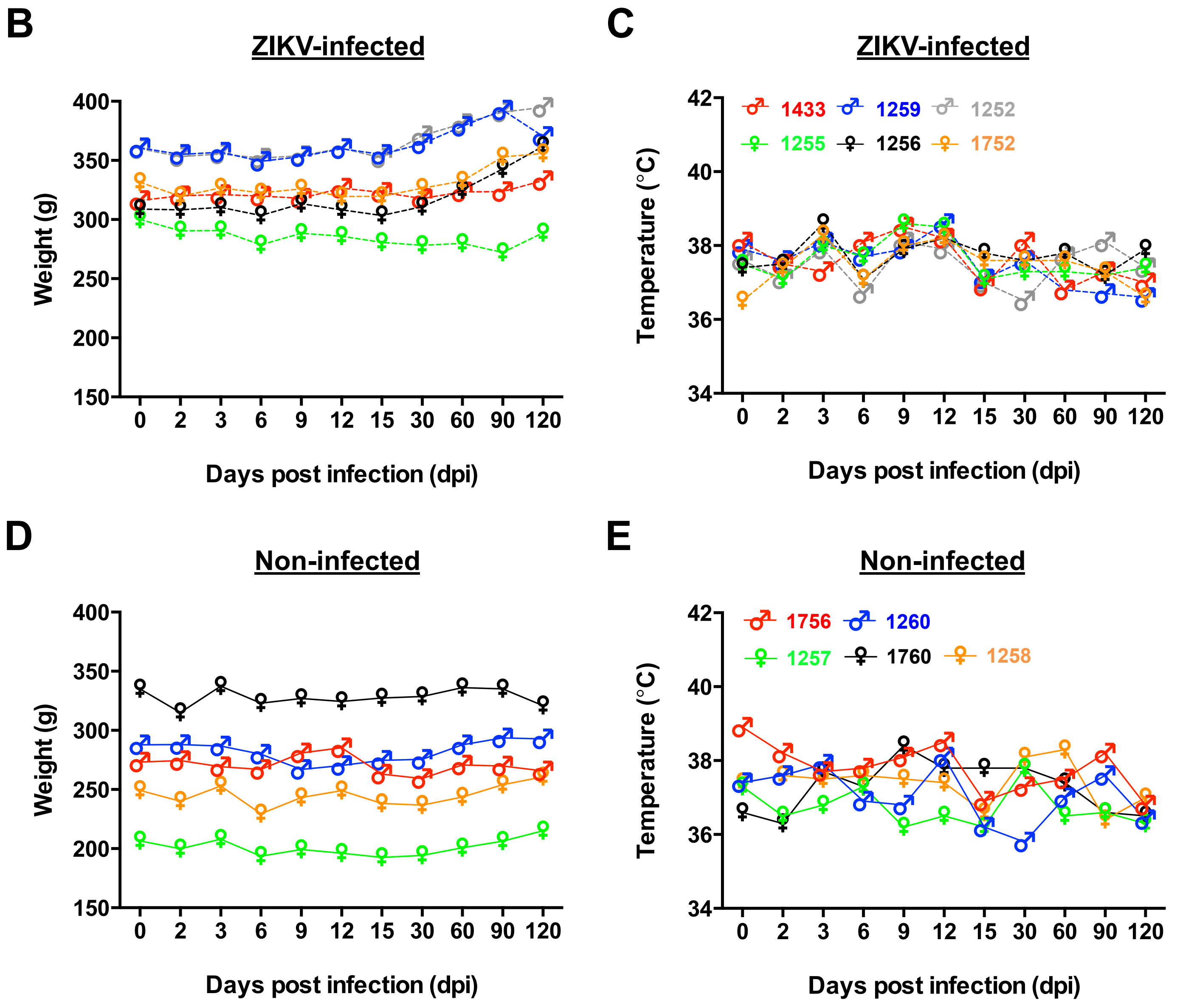

Figure S1: Assessment of physiological changes in experimental marmosets. (A) A total of 5 non-infected marmosets were used in parallel as negative controls. (B, D) weight and (C, E) temperature changes of (B, C) ZIKV-infected (n=6) or (D, E) non-infected marmosets (n=5) were determined at the stated time-points throughout the entire study. ZIKV-infected animals are depicted as dotted lines, whereas non-infected animals are depicted as continuous lines.

Figure S1 Lum et al., 2018

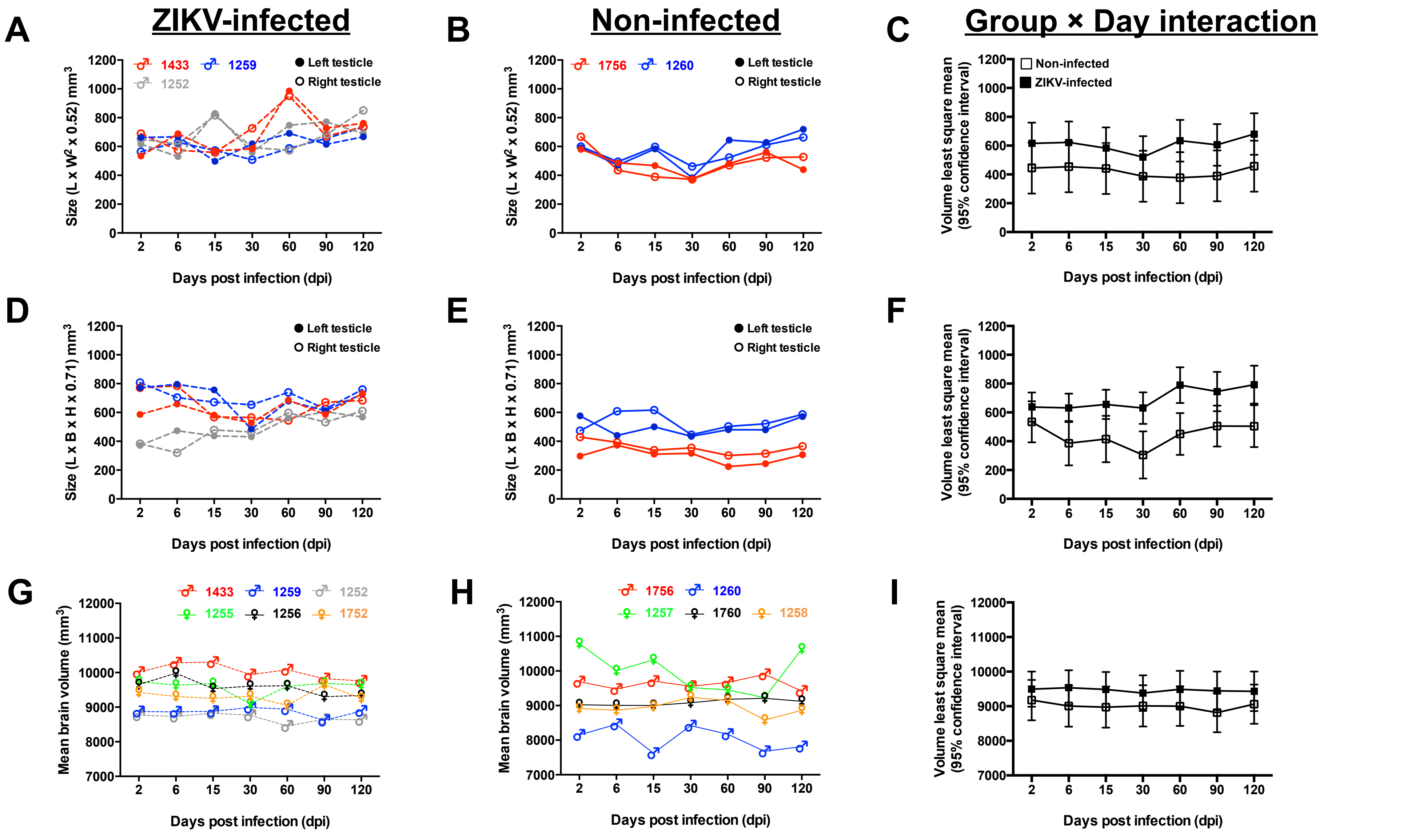

Figure S2: Detailing testicular and brain volumes in experimental marmosets. Mean testicular volumes were quantified (A–C) manually with vernier calipers or via (D–E) ultrasound imaging. (G–I) Mean brain volumes were quantified with magnetic resonance imaging. (C, F, I) Plots showing the volume least square mean of the testicular and brain volume measured throughout the study in both groups of marmosets after adjusting for differences in body weight (right column). ZIKV-infected (n=6) and non-infected (n=5) animals are reflected in the left and middle columns respectively.

Figure S2 Lum et al., 2018

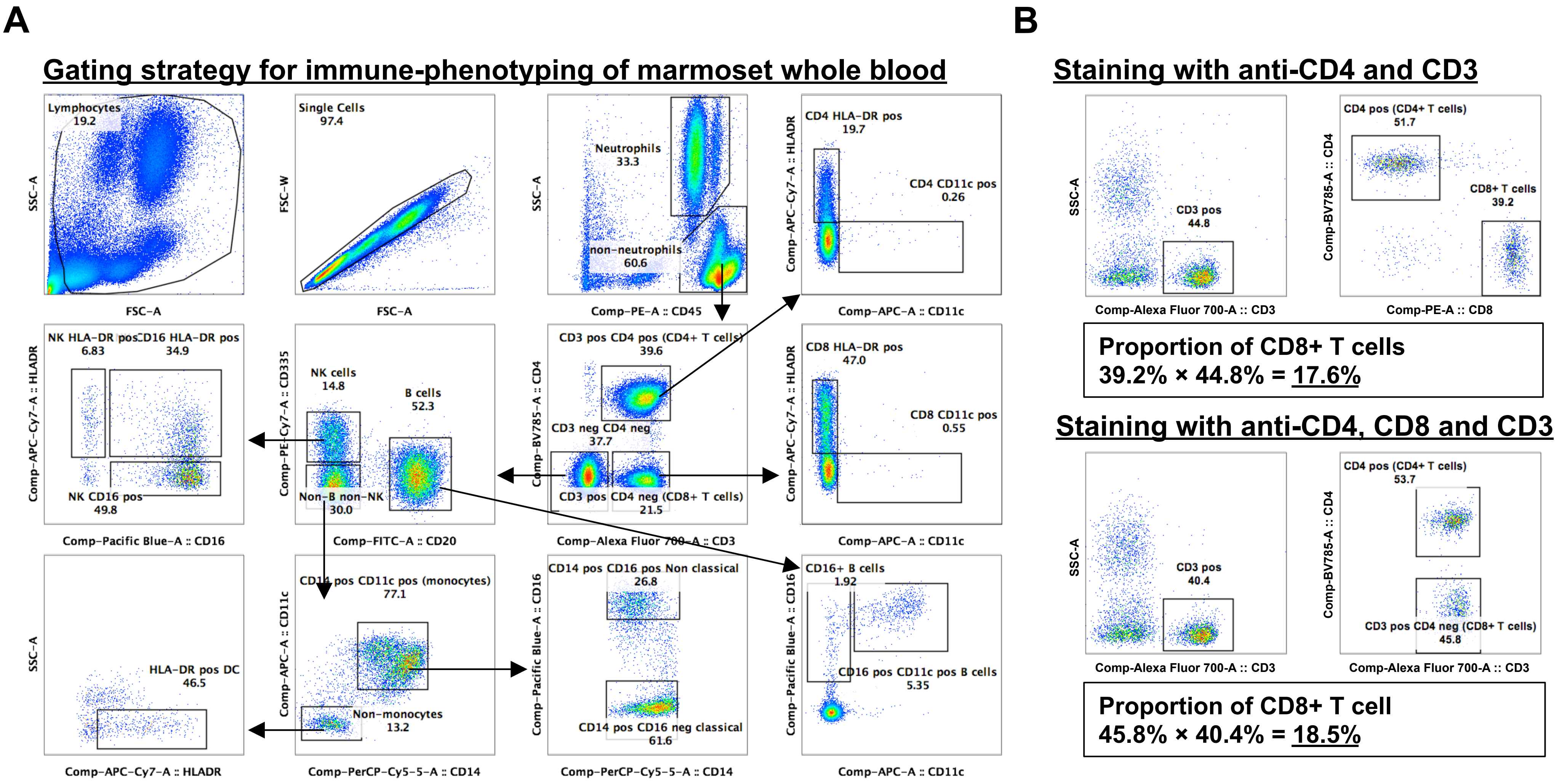

Figure S3: Gating strategy of marmoset whole blood immune-phenotyping. (A) Representative plots illustrating the gating strategy that identifies 15 unique immune populations with a panel of 9 antibodies. Neutrophils were first excluded with SSC-A. Non-neutrophils were first gated with CD4 and CD3 to identified the CD4+ and CD8+ T cells, which were further-gated with HLA-DR. Non-T cells were subsequently gated with CD335 and CD20 to identify the NK cells and B cells respectively. The NK cells were further characterized with CD16 and HLA-DR, while B cells were further characterized using CD11c and CD16. Monocytic cells were identified with CD11c, CD14 and CD16. Lastly, the DCs were gated from the non-monocytes with HLA-DR. (B) The efficacy of using only anti-CD4 and anti-CD3 antibodies in gating out CD8+ T cells. Percentages of CD8+ T cells of total gated cells were comparable between stainings without or with CD8-specific antibodies. The CD8 antibody was not included into the staining panel due to a clash of fluorophore wavelengths with the CD45 antibody.

Figure S3 Lum et al., 2018

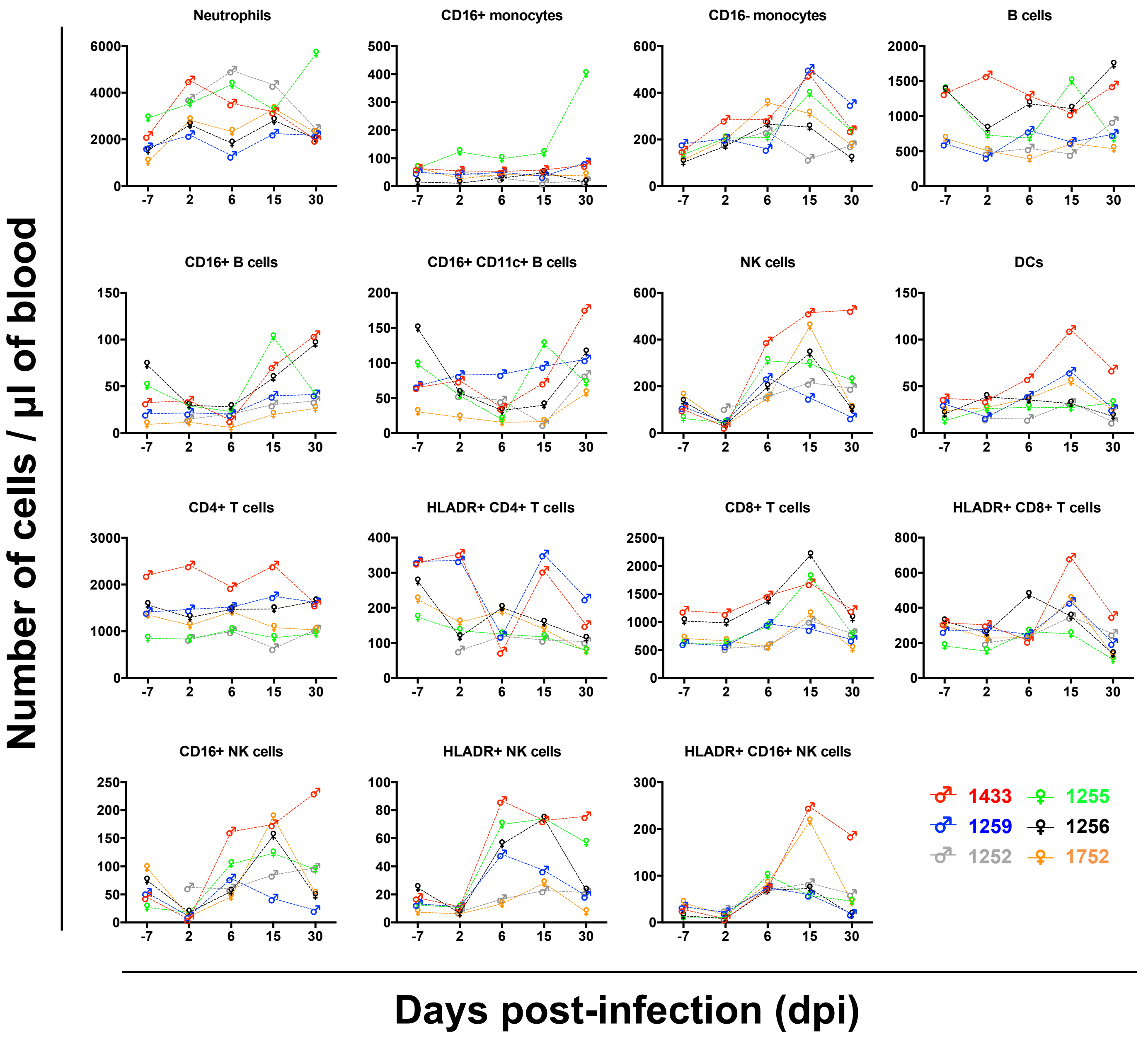

Figure S4: Immune-phenotyping of ZIKV-infected marmoset whole blood (n=6) was done from -7 to 30 dpi. Profiles of 15 immune populations (Neutrophils, CD16+ monocytes, CD16- monocytes, B cells, CD16+ B cells, CD16+CD11c+ B cells, NK cells, DCs, CD4 T cells, HLADR+ CD4 T cells, CD8 T cells, HLADR+ CD8 T cells, CD16+ NK cells, HLADR+ NK cells and HLADR +CD16+ NK cells) are shown. Values presented represent the number of cells per microliter of whole blood and is calculated by multiplying the percentages of each immune subset (from immune-phenotyping) with the total leucocyte numbers obtained with a hematology analyzer.

Figure S4 Lum et al., 2018

Supplementary Table 1: MRI acquisition scan parameters

Scan sequences	FOV (mm^2)	Number of slices (thickness mm)	TE (ms)	TR (ms)	Pixel Bandwidth (Hz/Px)	ETL/EPI factor	NEX	Pixel resolution (mm)	Flip angle (degrees)	Inversion Time, TI (ms)	Acquisition time (mm:ss)
Axial FSE	49 x 54	28 (1.0)	94	6470	186	12	3	0.2 x 0.2	165		6:35
Coronal FSE	49 x 54	28 (1.4)	94	6470	186	12	3	0.2 x 0.2	165		6:35
FLAIR	61 x 63	30 (1.0)	83	6460	289	16	4	0.2 x 0.2	150	2119	12:31
DWI[a]	113 x 113	13 (1.4)	50	2580	1068	60	1	1.9 x 1.9	180		4:15
MPRAGE	50 x 50	96 (0.4)	4.6	2250	140	112	3	0.4 x 0.4	9	900	12:36
MP2RAGE	50 x 50	96 (0.4)	4.2	4000	140	120	2	0.4 x 0.4	(i) 4<br>(ii) 5	(i) 793<br>(ii) 2510	14:56

[a] Additional parameters for readout-segmented DWI: 4-scan trace mode, with 9 readout segments and monopolar diffusion scheme. Two b-values: 0, 1800 s/mm^2, with 1 and 2 averages respectively.

Supplementary Table 2: Quantification of soluble immune mediators presence in plasma

	SDF-1α	IP-10	IL-6	IL-8	IL-10	Eotaxin	IL-12p70
One-way ANOVA *P* value	0.8902	0.4043	0.2195	0.6715	0.8335	0.7476	0.2825
One-way ANOVA *P* value (Multiple testing corrected)	0.8902	0.8495	0.8495	0.8495	0.8902	0.8495	0.8495
Dunnett's multiple comparisons test[a]							
ZIKV-infected 0dpi	0.9933	0.9681	0.9999	0.9999	0.9302	0.9999	0.9994
ZIKV-infected 2dpi	0.9998	0.5936	0.9643	0.9891	0.9997	0.9994	0.9742
ZIKV-infected 6dpi	0.9927	0.2906	0.9717	0.4380	0.9635	0.6748	0.3213
ZIKV-infected 14dpi	0.9908	0.7712	0.9538	0.8484	0.9997	0.9997	0.3385
ZIKV-infected 30dpi	0.9995	0.9995	0.9997	0.6655	0.9994	0.9473	0.9949
ZIKV-infected 60dpi	0.9996	0.7700	0.9994	0.9999	0.9998	0.9458	0.9996
ZIKV-infected 90dpi	0.9779	0.9998	0.0713	0.9751	0.9994	0.9070	0.9619
ZIKV-infected 120dpi	0.9998	0.9996	0.9442	0.9369	0.9757	0.9999	0.9969

[a] Analysis is compared to animal respective baseline sample obtained at 0dpi

IL-13	IL-1Rα	SCF	G-CSF	GM-CSF	TNF-α	MIP-1β	IFN-α	MCP-1	VEGF-D	MIG
0.8715	0.6781	0.1119	0.4999	0.6568	0.3387	0.6059	0.4818	0.0035	0.5749	0.5465
0.8902	0.8495	0.6994	0.8495	0.8495	0.8495	0.8495	0.8495	0.0738	0.8495	0.8495

0.9999	0.9788	0.9998	0.9972	0.9999	0.6538	0.6457	0.5528	0.9935	0.9403	0.9999
0.9996	0.9902	0.9921	0.9996	0.9995	0.3870	0.9885	0.5759	0.5964	0.9653	0.2854
0.9853	0.6992	0.7304	0.9999	0.9998	0.2957	0.2725	0.9998	0.9838	0.8890	0.9999
0.7346	0.9940	0.8371	0.9947	0.9868	0.7055	0.9924	0.3432	0.9891	0.9335	0.9995
0.9908	0.9999	0.1063	0.3793	0.7924	0.1348	0.5122	0.9999	0.0724	0.9949	0.9012
0.8384	0.6245	0.2192	0.7521	0.5237	0.1826	0.9712	0.3903	0.3465	0.9920	0.8990
0.9781	0.9794	0.0999	0.2815	0.3904	0.0611	0.7008	0.7453	0.2375	0.9994	0.9921
0.9949	0.9939	0.9250	0.8141	0.8947	0.3643	0.5568	0.9972	0.0450	0.9694	0.8536

bNGF	BDNF	IL-23	IL-15	IL-18	FGF-2	PDGF-ββ
0.5453	0.7214	0.0059	0.2557	0.4766	0.7306	0.0230
0.8495	0.8495	0.0738	0.8495	0.8495	0.8495	0.1917
0.9825	0.9904	0.1632	0.9997	0.9997	0.9705	0.9996
0.9999	0.9586	0.9968	0.9999	0.7349	0.7020	0.4270
0.4822	0.9999	0.9936	0.9950	0.4414	0.9996	0.1978
0.8641	0.9976	0.9720	0.9999	0.5784	0.6181	0.2492
0.9999	0.9999	0.6128	0.9947	0.9393	0.8975	0.0559
0.4622	0.9994	0.0475	0.7795	0.9978	0.9925	0.0097
0.7780	0.9185	0.7943	0.1323	0.3474	0.9997	0.0431
0.9996	0.9685	0.1513	0.9052	0.9393	0.9999	0.6269
